# Spindle Shaped Human Mesenchymal Stem/Stromal Cells from Amniotic Fluid Promote Neovascularization

**DOI:** 10.1371/journal.pone.0054747

**Published:** 2013-01-24

**Authors:** Maria G. Roubelakis, Grigorios Tsaknakis, Kalliopi I. Pappa, Nicholas P. Anagnou, Suzanne M. Watt

**Affiliations:** 1 Laboratory of Biology, University of Athens, Medical School and Cell and Gene Therapy Laboratory, Centre of Basic Research, Biomedical Research Foundation, Academy of Athens (BRFAA), Athens, Greece; 2 Nuffield Division of Clinical Laboratory Sciences, Radcliffe Department of Medicine, University of Oxford, and Stem Cell Research Laboratory, NHS Blood and Transplant, John Radcliffe Hospital, Headington, Oxford, United Kingdom; 3 First Department of Obstetrics and Gynecology, University of Athens, School of Medicine, Athens, Greece; National Cancer Institute, United States of America

## Abstract

Human amniotic fluid obtained at amniocentesis, when cultured, generates at least two morphologically distinct mesenchymal stem/stromal cell (MSC) subsets. Of these, the spindle shaped amniotic fluid MSCs (SS-AF-MSCs) contain multipotent cells with enhanced adipogenic, osteogenic and chondrogenic capacity. Here, we demonstrate, for the first time, the capacity of these SS-AF-MSCs to support neovascularization by umbilical cord blood (UCB) endothelial colony forming cell (ECFC) derived cells in both in vitro and in vivo models. Interestingly, although the kinetics of vascular tubule formation in vitro were similar when the supporting SS-AF-MSCs were compared with the best vasculogenic supportive batches of bone marrow MSCs (BMSCs) or human dermal fibroblasts (hDFs), SS-AF-MSCs supported vascular tubule formation in vivo more effectively than BMSCs. In NOD/SCID mice, the human vessels inosculated with murine vessels demonstrating their functionality. Proteome profiler array analyses revealed both common and distinct secretion profiles of angiogenic factors by the SS-AF-MSCs as opposed to the hDFs and BMSCs. Thus, SS-AF-MSCs, which are considered to be less mature developmentally than adult BMSCs, and intermediate between adult and embryonic stem cells in their potentiality, have the additional and very interesting potential of supporting increased neovascularisation, further enhancing their promise as vehicles for tissue repair and regeneration.

## Introduction

Mesenchymal stem/stromal cells (MSCs), first identified by Friedenstein et al. [1] in bone marrow, were subsequently found to contain multipotent cells capable of generating at least osteogenic, adipogenic and chondrogenic cells and of exhibiting immunomodulatory and stromal supportive properties for hematopoiesis [2]–[4] (reviewed in [5]–[9]). MSCs have since been described in a variety of tissues during development and in the adult, including amniotic fluid, umbilical cord, umbilical cord blood, bone marrow, placenta, adipose tissue and in the fetal circulation (reviewed in [5]–[9]) [10]–[17]. Since MSCs contain a heterogeneous mixture of both stem cells and their more differentiated progeny and since there is no single specific marker which defines the multipotent mesenchymal stem cell itself (reviewed in [6]), the MSC population has been defined by the International Society for Cellular Therapy as CD90^+^CD105^+^ CD73^+^ plastic adherent cells, lacking hematopoietic markers (e.g. CD45, CD19, CD14), but containing at least trilineage osteogenic, adipogenic and chondrogenic differentiation potential in vitro [18].

Amniotic fluid (AF) stem cells, which are reminiscent of adult bone marrow MSCs (BMSCs) in their plastic adherence, expression of such markers as CD90 and their lack of expression of hematopoietic lineage markers, are most frequent in the first trimester of pregnancy [19]–[24] (reviewed in [25]–[27]). In contrast to MSCs sourced post-natally, both these circulating fetal and second trimester AF- stem cell or AF-MSCs are reported to have increased proliferative potential, increased multipotentiality and longer telomeric lengths, but with AF-MSCs at earlier gestational stages expressing higher levels of endodermal and mesodermal markers than those at later gestational stages [21], [23], [24], [28]–[30](reviewed in [25]–[27]. Thus, the second trimester AF taken during scheduled amniocenteses is a rich source of multipotent MSCs.

AF-stem cells or AF-MSCs have been enriched using a variety of techniques, including one and two step cultures, CD117^+^ selection or short term culture to generate fibroblastoid colonies (reviewed in [26]) [19], [21], [23], [28], [29]. Using the latter approach, Roubelakis et al. [29] have identified and enriched for two subsets of human AF-MSCs, the spindle shaped (SS-AF-MSCs) and the round shaped (RS-AF-MSCs), obtained at the time of amniocentesis. These cells have distinctive morphologies, phenotypic differences and differing abilities to differentiate into multiple cell types. The spindle shaped amniotic fluid MSCs (SS-AF-MSCs) express pluripotency markers and have a greater propensity for adipogenic, osteoblastic and chrondrogenic differentiation [29]. Importantly, compared to adult human BMSCs, human AF-MSCs appear to be more stable karyotypically when cultured ex vivo [27], [30]. We have shown previously [29], [30], using the same SS-AF-MSC cell lines used in this manuscript, that karyotypic abnormalities could not be detected in at least these 6 cell lines when cultured for over 30–50 passages. It has been reported previously by some researchers that adult BMSCs can develop chromosomal abnormalities during in vitro culture and that this can lead to their malignant transformation[31]–[35], although, others have reported that human adult BMSCs do not malignantly transform in vitro and that aneuploidity may occur without transformation [36]. Since ageing is often associated with increased chromosomal abnormalities, increasing cancer rates and the acquisition of or exposure to viral infections, and generally a loss in the proliferative ability and the multipotency of MSCs [37], one advantage of sourcing cells from younger donors or at earlier stages of ontogeny might be that these risks or disadvantages are reduced, thus making them a preferred source of cells. Indeed, in our publications [29], [30], we have shown that SS-AF-MSCs can be expanded significantly more in culture than BMSCs and that this occurs without karyotypic changes and with maintenance of their potency for generating osteogenic, adipogenic and chondrogenic cells. In addition, when compared to perinatal or postnatal MSCs, none of the AF-MSC cell lines tested so far has exhibited a susceptibility to transformation in culture nor have they generated teratomas in vivo [11], [23], suggesting that AF-MSCs may constitute a potentially safe prospective tool for clinical applications as supported by our recent publication in disease mouse models [37].

We have shown that human dermal fibroblasts (hDFs) and BMSCs support vascular tubule formation from umbilical cord (UC) and umbilical cord blood (UCB) endothelial colony forming cells (ECFC) in vitro [38]–[41]. However, the ability of AF-MSCs to support neovascularization has not been addressed. Here, we show, for the first time, that the highly proliferative human SS-AF-MSCs produce a defined profile of angiogenic factors, and that they have similar capacities to support neovascularization by UCB ECFC derived cells in vitro as the best pre-selected vasculogenic supportive batches of adult BMSCs or hDFs. However, in vivo, human SS-AF-MSCs are much more effective than BMSCs in supporting neovascularization in vivo in NOD/SCID mice.

## Materials and Methods

### Human Umbilical Cord Blood Endothelial Colony Forming Cell (ECFC) Derived Cells

Umbilical cord blood (UCB) units were sourced and collected with informed written consent from the John Radcliffe Hospital in Oxford with ethical approval for collection from the Oxford Research Ethics Committee and used in these studies with ethical approval from the Berkshire Research Ethics Committee as described previously [17], [39], [42] and with approval of the NHSBT R&D institutional review committee. UCB mononuclear cells (MNC) were isolated by density gradient centrifugation using Ficoll-Histopaque (density 1.077 g/ml; PAA Laboratories GmbH, Pasching, Austria) and 2×10^7^ MNC/well were seeded into 6-well plates pre-coated with 50 µg/ml type I rat tail collagen (BD Biosciences, Aylesbury, England) in endothelial growth medium-2 (EGM-2; Lonza Biologics, Tewkesbury, England) containing 10% (v/v) Hyclone fetal calf serum (FCS; Fisher Scientific, Loughborough, England; termed complete EGM-2 medium). After 24 hours of culture, non-adherent cells were removed by washing with EGM-2 medium and adherent cells then cultured in complete EGM-2 medium at 37°C, 5% CO_2_ in a humidified incubator [39], [42], [43]. The medium was changed daily for seven days and then every other day until the first passage. Single colonies derived from ECFCs appeared between 5 and 14 days of culture as well circumscribed cobblestone monolayers as described [40], [44]. Those with ≥50 cells were trypsinized by the end of this 2 week culture period using polystyrene cloning rings (Sigma-Aldrich Company Ltd., Poole, England), and colonies from single UCB units pooled, and plated into 25 cm^2^ tissue culture flasks pre-coated with type I rat tail collagen in complete EGM-2 medium. Cells from this pool when they reached confluency 1–2 days later were termed passage (p) 1 ECFC derived cells.

### eGFP-lentiviral Vector Generation, Production and Transduction of ECFC Derived Cells

Where indicated, UCB ECFC derived cells were transduced with the second generation self-inactivating amphotrophic HIV-based lentiviral vector expressing enhanced-green fluorescent protein (eGFP) under the control of the spleen focus forming viral (SFFV) promoter, a kind gift from Professor Adrian Thrasher and as previously described [39]. The eGFP lentiviral vector was propagated and packaged by transient three plasmid (50 µg eGFP lentiviral genome, 17.5 µg vesicular stomatitis virus-G (VSV-G) and 32.5 µg D8.91 plasmids) co-transfection in 293 T cells using Lipofectamine 2000 as recommended by the manufacturer (Invitrogen Ltd., Paisley, Scotland) instead of polyethyleneimine (PEI). The crude vector containing medium was collected from cells 48 h post transfection and titered on 293 T cells. Viral stocks propagated in this way were within the range of 1.5 × 10^6^ to 2 × 10^7^ Infectious Units (IU)/mL. Optimization of the transduction efficiency was similar to that described for UC ECFC derived cells. UCB ECFC derived cells were subsequently labeled with eGFP by transduction with viral vectors at multiplicities of infection (MOIs) ranging from 7 to 15 with a transduction efficiency of approximately 100% and with no significant loss of cell viability as described [39].

### Amniotic Fluid Mesenchymal Stem/Stromal Cells (AF-MSCs)

Cultured AF-MSCs were isolated from 3 human AF samples, collected during scheduled normal pregnancy amniocentesis between the 15^th^ and 18^th^ weeks of gestation, as described previously [29], [30], [37], [45]. All second trimester amniotic fluid samples were obtained with written informed consent, approved by the Ethical Committee of the Alexandra Hospital and the Bioethics Committee of the University of Athens School of Medicine. All samples used were derived from the excess volume of amniotic fluid taken for prenatal diagnosis. Using a 22G needle and under ultrasonographic control, 10–15 ml of amniotic fluid was aspirated for each sample. The procedure-related spontaneous abortion rate ranges from 0.06 to 0.5% [46], [47]. Each sample was centrifuged at 1,300 rpm for 10 min. The pellet was resuspended in Dulbecco’s modified Eagle’s medium (DMEM; Sigma-Aldrich Company Ltd.) supplemented with 20% (v/v) FCS (Gibco-BRL, Paisley, Scotland) in a 25 cm^2^ tissue culture-treated flask and incubated at 37°C in a 5% humidified CO_2_ chamber for approximately 8–12 days, when the first colonies appeared. Spindle shaped (SS) colonies were selected at passage 0 and cells were subcultured as described previously [29]. SS-AF-MSCs were used by passages 5–12.

### Human Dermal Fibroblasts (hDFs)

Normal hDFs were purchased as passage (p) 1 from Lonza Biologics and cultured in Dulbecco’s modified Eagle’s medium (DMEM) with high glucose and sodium pyruvate, supplemented with L-glutamine, penicillin plus streptomycin (Invitrogen Ltd.) and 10% (v/v) FCS (PAA Laboratories GmbH) [40], [41]. Selected batches of hDFs, which optimally supported vessel formation in vitro, were used at passages 5–6.

### Bone Marrow Mesenchymal Stem/Stromal Cells (BMSCs)

Bone Marrow mesenchymal stem/stromal cells (BMSCs) were purchased from Lonza Biologics at passage 2 and cultured in mesenchymal stem cell growth medium (MSCGM) according to manufacturer’s protocols [17], [38], [39]. Selected batches of BMSCs, which optimally supported vessel formation in vitro, were used at passages 5–6 in all experiments.

### Cell Surface Phenotype of Umbilical Cord Blood (UCB) ECFC Derived Cells

UCB ECFC derived cells (p3–5) were incubated with Fc receptor blocking agent (Miltenyi Biotec., Bergisch Gladbach, Germany), followed by relevant conjugated monoclonal antibodies (Mabs) or isotype-matched negative controls [38], [39]. The following mouse Mabs were used: phycoerythrin conjugated (PE)-CD31 (mIgG1; clone L133.1), PE-CD73 (mIgG1; clone AD2), PECy7-CD14 (mIgG2a; clone M5E2), PECy7-CD45 (mIgG1, clone HI30), as well as the isotype controls: PECy7-mIgG2a, PE-, PECy7- and fluorescein isothiocyanate (FITC)-mIgG1, APC-mIgG2a) (all from BD Biosciences); PE-CD133 (mIgG2b, clone 293C3) from Miltenyi Biotec.); PE-mIgG2b isotype control (clone 133303) from R&D Systems (Abingdon, England); FITC-CD146 (mIgG1; clone P1H12) from Millipore Ltd., Bedford, MA, USA; FITC-CD105 (mIgG1; clone 166707), PE-CD144 (clone 16B1; mlgG1, E-Bioscience, Hatfield, England) APC-CD34 (clone BIRMA-K3, mlgG1, DakoCytomation, Glostrup, Denmark). Cells were analysed on a BD LSR II flow cytometer using FACSDiva software (BD Biosciences) as previously described [34], [42], [43]. Cell viability was determined as negative staining for the Topro-3 dye (Invitrogen Ltd.). Results for the phenotypic analyses of the UCB ECFC derived cells are presented in Figure S1. Umbilical cord ECFC derived cells were CD31^+^CD73^+^CD105^+^ CD146^+^CD144^+^CD45^−^CD14^−^CD133^−^ and displayed variable CD34 levels in culture, confirming their endothelial lineage phenotype.

### Proliferation Assays in Presence of SU6668 Inhibitor

UCB ECFC derived cells, SS-AF-MSCs, BMSCs and hDFs were plated at a density of 1.5×10^4^/ml in a 96 well plate and were cultured for 3 and 8 days in the presence of DMEM (10% (v/v) FCS) or DMEM (10% (v/v) FCS) supplemented with 5 µΜ or 10 µΜ SU6668 inhibitor (Calbiochem, Merck Chemicals Ltd., Nottingham, England) in 5 replicates for each concentration. After 3 and 8 days, CellTiter 96 AQueous One Solution cell proliferation assays (MTS) (Promega Ltd., Madison, WI, USA) were performed. The absorbance was recorded at 490 nm using a microplate reader (ELX 800, Biotek Instruments Inc, VT, USA). Results were expressed as the percentage of proliferation increase, calculated using the following formula: [(OD_dayx_-OD_day0_)/(OD_control_ - OD_day0_)x100], where OD_control_ corresponded to the absorbance measured in non treated cells on day x. All assays were performed in triplicates and the mean of each experiment was calculated. Statistical analysis was performed using Student’s *t* test.

### Proliferation Assays in Presence of Conditioned Media Containing IL-8 and PDGF-AB/BB Neutralizing Antibodies or MMP9 Inhibitor

For the preparation of the conditioned media (SS-AF-MSC-CM), 1×10^6^ SS-AF-MSCs were cultured until 80% confluent in DMEM with 20% (v/v) FCS, and then the medium was replaced with EBM-2 medium containing 0.5% (v/v) FCS to prevent protein aggregation. Cells were cultured for a further 24 hours and the CM was collected and concentrated approximately 25-fold using ultra filtration units with a 3-kD cut-off (Millipore Ltd.). Sham medium comprised EBM-2 medium containing 0.5% (v/v) FCS which had been incubated for 24 hours in the absence of cells. For proliferation assays, UCB ECFC derived cells were plated in complete EGM-2 medium in 96-well plates (Corning Life Sciences Ltd.) and incubated overnight at 37°C in a humidified air 5% CO_2_ incubator. The following day, media were replaced by conditioned or sham media diluted in EGM-2 medium (1∶1). On day 3, MTT (Sigma-Aldrich Company Ltd.) was added to each well and absorbance at 570 nm was then measured. For blocking experiments, polyclonal rabbit anti-human IL-8 (Peprotech EC Ltd., London, England, 5 µg/ml) and PDGF-AB/BB mouse anti-human PDGF-AB/BB (BD Biosciences, 5 µg/ml) antibodies (Abs) or MMP-9 inhibitor (Santa Cruz Biotechnology, Inc Santa Cruz, CA, USA, 15 µM) were added in the SS-AF-MSC-CM at day 0.

### Transwell Migration Assay-in vitro Blocking Experiments

In vitro motility assays were performed as previously described [48]. In brief, UCB ECFC derived cells at passage 2–6 were transferred at 2×10^4^/100 µL density to the insert of a transwell plate with 8 *µ*m pore size (Corning Life Sciences Ltd.) in EBM-2: EGM-2 (4∶1) medium. Cells were then allowed to migrate for 6 h across the pore membrane, toward Sham medium or SS-AF-MSC-CM. After the 6 h incubation period, the non-migrated cells were removed from the top of the insert with a wet cotton swab. The migrated cells were then fixed with 4% (w/v) paraformaldehyde on the membrane and stained sequentially with eosin and hematoxilin (all from Sigma-Aldrich Company Ltd.). Migration was quantified by counting the nuclei that passed through the filter. Photographs of the stained nuclei were taken from a minimum of 10 fields of view (20Χ) for each membrane using an inverted TE300 microscope (Nikon Ltd., London, England) fitted with a cooled CCD camera and Simple PCI software (Digital Pixel, Brighton, England) and experiments were performed repeated twice. Statistical analysis was performed using Student’s *t*-test. For the in vitro blocking experiments, IL-8 and PDGF-AB/BB were blocked in SS-AF-MSC-CM with the respective neutralizing Abs described above at a concentration of 5 µg/ml for 10–30 min at 4°C. CM was also incubated under the same conditions with irrelevant control Abs. For MMP-9 blocking experiments, the MMP-9 inhibitor-I (15 µM) was added in SS-AF-MSC-CM. Fresh EGM-2 medium and CM derived from HEK-293 T cells were used as control media. In addition, recombinant IL-8 (Peprotech EC Ltd.) or PDGF-AB/BB (Peprotech EC Ltd.) were added, both at 1 µg/ml, in EGM-2 medium supplemented with 0.5% (v/v) FCS.

### Vascular Tubule co-culture Assay

For tubule formation, 3 batches of UCB ECFC derived cells at p4–6 were seeded with SS-AF-MSCs, and pre-selected batches of BMSCs or hDFs known to be good supporters of vessel formation in vitro in a coculture assay. In initial experiments, SS-AF-MSCs, BMSCs and hDFs (ranging from 1×10^4^ to 4×10^4^ per well) were plated with UCB ECFC derived cells (ranging from 1×10^3^ to 1× per well) in 48-well collagen-I coated plates to determine the best ratio and cell numbers required for optimum tubule formation. A ratio of 1∶4 ECFC derived cells: MSCs or hDFs was estimated as the optimum for the co-culture experiments. Subsequently, SS-AF-MSCs, BMSCs (p5–6) or hDFs (p5–6) were plated in complete EGM2 medium in 48-well plates [38], [41], the culture plate gently swirled to evenly distribute the SS-AF-MSCs, BMSCs or hDFs and cells were incubated for 2 to 24 h at 37°C and 5% CO_2_. eGFP labeled UCB ECFC (p4–6) were detached with trypsin-EDTA, counted, resuspended in complete EGM-2 medium and added to the wells with the preplated SS-AF-MSCs, BMSCs or hDF. The plates were gently swirled, incubated at 37°C and 5% CO_2_, the medium was changed every 2–3 days and the cells were cultured for up to 14 days at which point the cells were fixed, and eGFP-expressing tubules were photographed at 4× magnification on an inverted TE300 microscope (Nikon Ltd.) fitted with a cooled CCD camera and Simple PCI software (Digital Pixel). Images were processed in Adobe Photoshop 7 and tubule numbers, lengths and number of junctions were quantified using Angiosys software (TCS Cellworks, Buckingham, England). To test the sensitivity of vascular tubule development to anti-angiogenic factors, SS-AF-MSCs, BMSCs or hDFs were co-cultured with UCB ECFC derived cells and SU6668 inhibitor (at 5 and 10 µM in DMSO; Calbiochem) [42] was added to co-cultured cells in triplicate wells in complete EGM-2 medium at the initiation of the culture. Fresh SU6668 was also added at each medium change (3–4 day intervals) for the duration of the assay over 14 days.

### Matrigel Assay

UCB ECFC derived cells were trypsinized and resuspended in eight different types of media as follows: (1) SS-AF-MSC-CM; (2) SS-AF-MSC-CM+MMP-9 inhibitor (Santa CruzBiotechnology, Inc., 15 µM); (3) SS-AF-MSC-CM+anti-human IL-8 Ab (Peprotech EC Ltd., 5 µg/ml); (4) SS-AF-MSC-CM+anti-human PDGF-AB/BB Ab (BD Biosciences, 5 µg/ml); (5) complete EGM-2 medium; (6) EBM-2+0.5% (v/v) FCS; (7) EBM-2+0.5% (v/v) FCS+recombinant (rec) IL-8 (Peprotech, 1 µg/ml); and (8) EBM-2+0.5% (v/v) FCS+recombinant (rec) PDGF-AB/BB (Peprotech, 1 µg/ml). For each condition, UCB ECFC derived cells were plated at a density of 1.5 x 10^4^ cells/well in triplicate in 96-well plates coated with 50 µL of growth factor-reduced Matrigel (BD Biosciences) [42]. Plates were incubated for 20 h at 37°C before photomicroscopy. After incubation, media were removed, plates washed with distilled water (PAA Laboratories GmbH) twice, and cells were fixed using 100% (v/v) ice-cold methanol (Sigma-Aldrich Company Ltd.). Each image from each well was taken at 4 magnification using a Nikon Eclipse TE2000-U microscope (Nikon Ltd.).

### Tubule Analysis Using Angiosys Software

Quantitation of the vascular tubules was performed by photographing eGFP-expressing tubules at 4× magnification on an inverted TE300 microscope (Nikon Ltd) fitted with a cooled CCD camera and Simple PCI software (Digital Pixel). Images were processed in Adobe Photoshop 7 and image analysis of tubule numbers, lengths and number of junctions were quantified using Angiosys software (TCS Cellworks) as described [41], [42]. For quantification, cells were cultured in triplicate, then 4 central fields/well were analyzed from each assay well. Results for the different groups assayed are expressed as means ±S.E.M.

### Proteome Profiler Arrays of Conditioned Media (CM)

For the preparation of the CM, 1×10^6^ SS-AF-MSCs, BMSCs, UCB ECFC or hDFs were cultured until 80% confluent in their respective media, and then the media were replaced with EBM-2 medium containing 0.5% (v/v) FCS to prevent protein aggregation. The cells were cultured for a further 24 hours and the CM was collected and concentrated approximately 25-fold using ultra filtration units with a 3-kD cut-off (Millipore Ltd. Ltd.), and analyzed for specific proteins using proteome profiler arrays for angiogenesis growth factors (Catalog # ARY007, R&D Systems Inc., Minneapolis, USA) according to the manufacturer’s instructions. Quantitation of the detected spots was performed using the Quantity One Software 4.4.1 (BioRad Laboratories Inc., Amersham, England). Three different samples for each cell type were tested, each in two replicates. Results for the different growth factors assayed are expressed as means ±S.E.M and statistical analysis was performed using Student’s *t* test.

### Matrigel in vivo Plug Assay

NOD-SCID mice were housed and maintained at the Animal Facility of the Biomedical Research Foundation of the Academy of Athens (BRFAA). The procedures for the care and treatment of animals were performed according to the institutional guidelines which follow the guidelines of the Association for Assessment and Accreditation of Laboratory Animal Care (AAALAC) and the recommendations of the Federation of European Laboratory Animal Science Associations (FELASA) and approved by the Institutional (BRFAA) Animal Care and Use Committee. For the in vivo assays, 10^6^ unlabeled or eGFP UCB ECFC derived cells combined with SS-AF-MSCs, BMSCs or hDFs at a ratio of 4∶1 (ECFC derived cells: MSCs or hDFs), or UCB ECFC derived cells only, or SS-AF-MSCs, BMSCs or hDFs only were suspended in 200 µl of matrigel (cat. no. #356237, basement membrane matrix phenol red-free, BD Biosciences) and injected into the back of the 6–8 week old NOD-SCID mice in a slightly modified protocol to those described [49]. Six animals per group were used. For the negative control, 200 µl of matrigel was injected without added cells. The animals were sacrificed 2 weeks later and the matrigel mass was excised. Ten to twenty minutes before sacrifice 3 animals per group were intravenously (tail vein) injected and perfused with 200 µl biotinylated tomato lectin (1∶20) (Vector Laboratories, Peterborough, England).

### Immunofluorescence on Cryostat Sections

Matrigel masses were collected and fixed in 4% (w/v) paraformaldehyde (Sigma-Aldrich Company Ltd.) and then 5 µm matrigel cryosections were prepared following standard protocols. Nonspecific binding was blocked using 5% (v/v) normal rabbit serum (Gibco-BRL) in PBS with 0.1% (v/v) Triton-X (Sigma-Aldrich Company Ltd.) for 30 min. Sections were subsequently incubated with mouse anti-human CD31 (hCD31; clone JC70A; BD Biosciences, 1∶50 dilution**)** and/or rat anti-mouse CD31 (mCD31; MEC 13.3; BD Biosciences, 1∶50 dilution**)** primary antibodies overnight at 4°C. For negative controls, appropriate isotype antibodies were used. Sections were then incubated with goat anti-mouse Alexa 568 or goat anti-mouse Alexa 488 and/or goat anti-rat Alexa 647 secondary antibodies (Invitrogen Ltd.) for 1 h at room temperature. Tomato lectin was detected with streptavidin-conjugated Alexa 546 (Invitrogen Ltd.). 4,6-diamino-2-phenylindole (1∶1000.) (DAPI, Sigma-Aldrich Company Ltd.) was used to visualize the cell nuclei. Slides were mounted with fluorescent mounting medium (DakoCytomation Ltd., Glostrup, Denmark) and visualized under an automated Leica TCS SP5 confocal microscope fitted with HeNe 543, HeNe 633 and argon 488 lasers (Leica Microsystems GmbH Wetzlar, Germany).

### Immunohistochemistry, Vessel Quantitation and Diameter Estimation

Matrigel implants were removed at two weeks after implantation, fixed in 4% (w/v) paraformaldehyde (Sigma-Aldrich Company Ltd.) and sectioned as 5 µm cryosections. Sections were incubated with mouse anti-human CD31 monoclonal (DakoCytomation) or rat anti-mouse CD31 monoclonal (BD Biosciences) primary antibodies. Sections were then incubated with biotinylated goat anti-rat (BD Biosciences) or goat anti-mouse secondary antibodies (DakoCytomation) for 30 min, followed by ABC-complex-HRP (DakoCytomation) for another 30 min. Reactions were developed for 5 min in DAB (Vector Laboratories), counterstained in Gill’s hematoxylin (Sigma-Aldrich Company Ltd.), subsequently dehydrated, mounted and studied by light microscopy. Alternatively, hematoxylin and eosin (H&E) staining was performed to examine the presence of vascular structures containing red blood cells. Images were taken with a Leica CTR MIC microscope and then the number of vessels per mm^2^ and the diameter of each vessel (in microns) were estimated using the Image J 1.38× software (http://rsb.info.nih.gov/ij/). Statistical analysis was performed using Student’s *t* test, (*p<0.05). A minimum of 15 fields of view (at 40× magnification) was analyzed from each photograph. Error bars indicate S.D. of the mean for 10 photographs from each group.

## Results

### Cell Characterization

In the present study, we used batches of SS-AF-MSCs and BMSCs which had been characterized previously in terms of their phenotype and ability to give rise to adipogenic, chrondrogenic and osteogenic lineages [17], [29], [30]. The SS-AF-MSCs and BMSCs used in these experiments expressed high levels of CD73, CD90, CD105, CD29, CD44 and CD166 surface markers, but lacked hematopoietic surface markers such as CD45 and CD14 and hematopoietic/endothelial markers such as CD31 when analyzed by flow cytometry [17], [29], [30], thereby confirming their MSC phenotype. The endothelial lineage phenotype of UCB ECFC derived cells was confirmed as described in the Supplementary data with cells lacking CD90, as well as the hematopoietic markers, CD45 and CD14, but expressing CD31, CD73, CD105, CD144 and CD146 (Figure S1).

### SS-AF-MSCs have a Similar Capacity to BMSCs and hDFs in Supporting Vascular Tubule Formation in vitro

**Figure 1:**
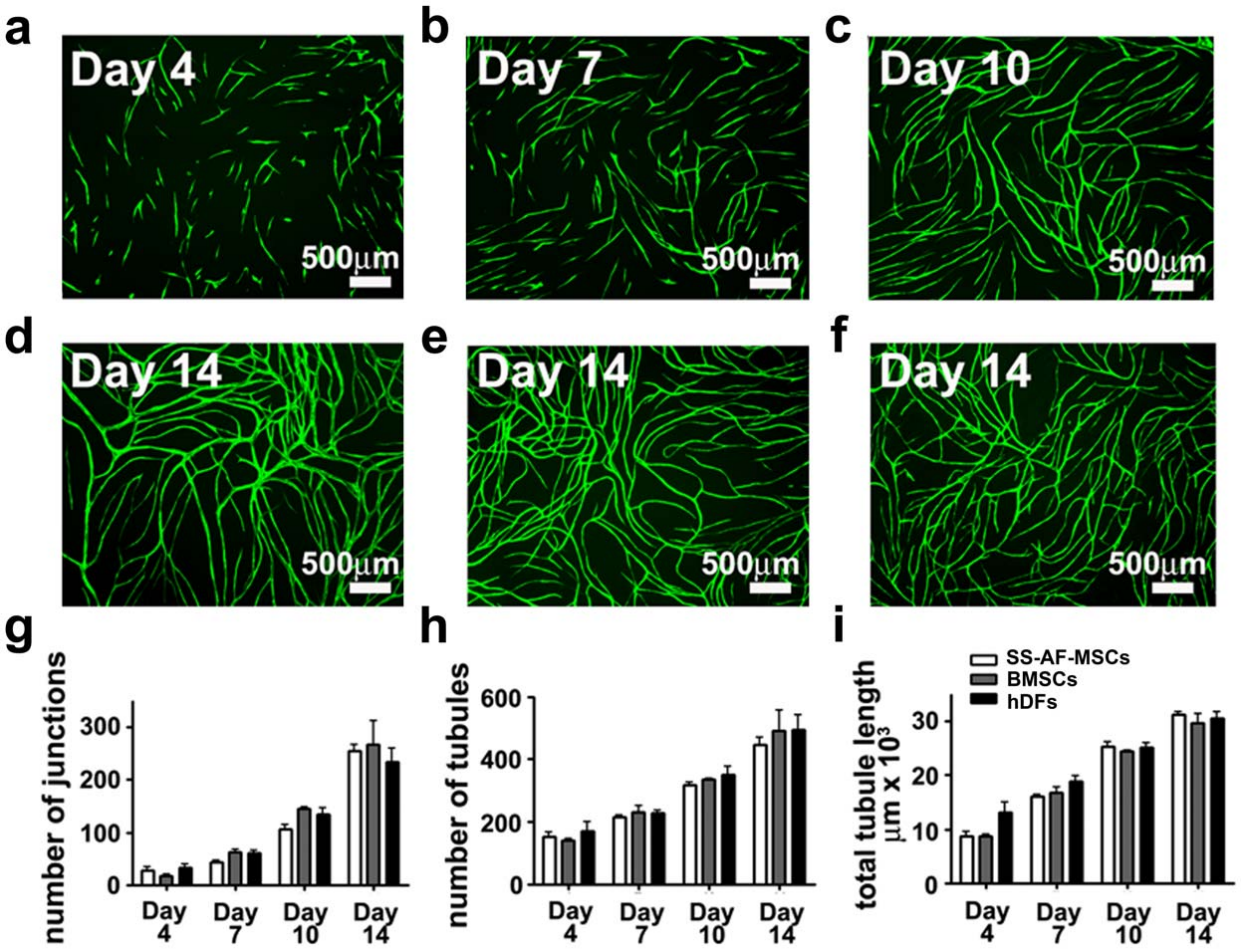
SS-AF-MSCs support neovascularisation in vitro – comparing the dynamics of vessel formation in the presence of SS-AF-MSCs, hDFs or BMSCs. (a-f) Representative fields of vascular tubules after (a) 4, (b) 7, (c) 10, and (d to f) 14 days of co-culture of eGFP UCB ECFC derived cells with (a to d) SS-AF-MSCs, and pre-selected optimized batches of (e) BMSCs or (f) hDFs respectively. Scale bar = 500 µm. (g-i) Quantification of vascular tubule phenotypes at days 4, 7, 10 and 14, showing no significant difference in the number of junctions, tubules and total tubule length during the 14 days of co-culture between the 3 stromal cell types (p>0.05 Student’s *t* test). Values are means±S.D. for three independent experiments.

We initially examined the ability of SS-AF-MSCs, to support vascular tubule formation in vitro, comparing these to pre-selected batches of BMSCs and hDFs which optimally supported vessel formation. Figure 1 (a to d) shows that eGFP-labeled UCB ECFC derived cells co-cultured with SS-AF-MSCs formed vascular tubule networks over the course of 2 weeks of culture. When UCB ECFC derived cells were plated together with SS-AF-MSCs (Figure 1 a to d) or optimal batches of hDFs (Figure 1 f) or BMSCs (Figure 1 e), they initially adhered to collagen 1 coated dishes. By day 4, vascular tubule development was obvious and the tubule network formation was characterized by intense sprouting over the next 6 days of culture (Figure 1 a to c; days 4 to 10; data not shown). This was followed by consolidation and the formation of an anastomosing network of tubules (Figure 1d; day 14).

Quantitation of vascular tubule formation by UCB ECFC derived cells in the presence of supporting stromal cells (Figure 1 g to i) revealed that, over the course of 14 days of co-culture, the dynamics of vascular tubule formation by UCB ECFC derived cells in the presence of SS-AF-MSCs did not significantly differ from that observed in the presence of the optimal batches of BMSCs or hDFs based on the number of junctions or tubules formed, or the total tubule length. SS-AF-MSCs supported the formation of networks with 254±16 junctions, 445±31 tubules and a total tubule length of 31182±728 microns, compared to 267±55 and 235±32 junctions, 490±80 and 494±58 tubules, and total tubule lengths of 29702±2107 and 30571±1452 microns for BMSCs and hDFs respectively (p>0.05, SS-AF-MSCs compared to BMSCs or hDFs). We next chose to add SU6668, a competitive angiogenesis inhibitor, which disrupts the integrity of the association of stromal cells or pericytes with endothelial cells [42], [50]–[53] to our UCB ECFC-AF-MSC co-cultures in vitro to confirm the efficacy of vascular tubule formation in vitro. SU6668 (5 µM and 10 µM) when added from the start of and then chronically throughout the co-cultures severely disrupted vascular tubule formation in vitro in a concentration dependent manner, with the number of vascular junctions, tubules and total tubule length being significantly different with SU6668 addition than without it (p<0.05 Student’s *t* test; n = 3 independent experiments; Figure S2). This SU6668 treatment did not affect the proliferation of SS-AF-MSCs, BMSCs, hDFs or UCB ECFs as shown by the MTS proliferation assay 3 and 8 days after treatment (Figure S3).

### SS-AF-MSCs Support in vivo Vessel Formation more Effectively than BMSCs

**Figure 2:**
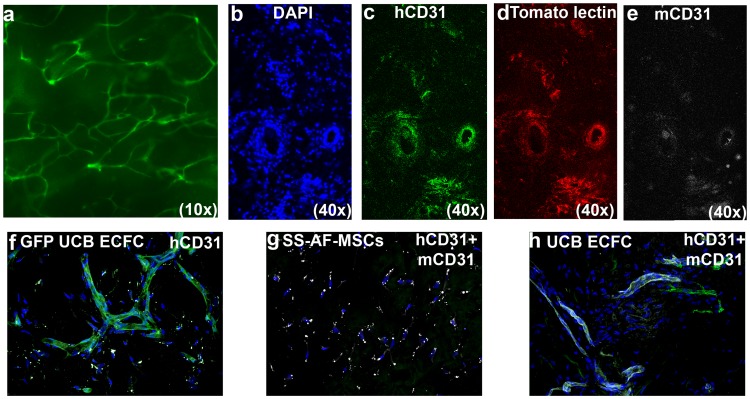
Immunofluorescence vessel imaging. (a) Representative photomicrographs of the matrigel implants containing eGFP-UCB ECFC derived cells and SS-AF-MSCs. (b-e) Representative photomicrographs of matrigel implant sections containing non eGFP tagged UCB ECFC derived cells and SS-AF-MSCs after staining with (b) DAPI (blue), (c) hCD31 (green), (d) following tomato lectin perfusion (red), and (e) mCD31 (white). (f) Co-localization of eGFP (green) with hCD31 (white) staining in matrigel implant sections containing eGFP-UCB ECFC derived cells and SS-AF-MSCs. (g) Representative photomicrographs of matrigel implant sections containing SS-AF-MSCs only stained for hCD31 (green) and mCD31 (white) antigens, but where hCD31 was not detected. (h) Representative photomicrographs of matrigel implant sections containing UCB ECFC derived cells only, stained for hCD31 (green) and mCD31 (white) antigens.

**Figure 3:**
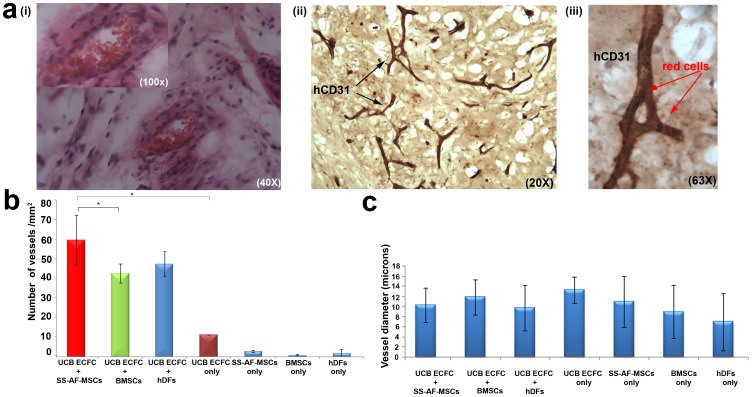
Quantitating vessel formation in in vivo studies. (a) Histological evaluation of vessels containing SS-AF-MSCs and UCB ECFC derived cells, harvested 14 days post-implantation and stained (i) with hematoxylin/eosin and for (ii-iii) human CD31 antigen (brown stain). High-power view of a vessel containing red blood cells (arrowed) from (ii) is shown in (iii). (b) Microvessel density in matrigel implants containing combined SS-AF-MSCs, BMSCs or hDFs with UCB ECFC derived cells, SS-AF-MSCs only, BMSCs only, hDFs only or UCB ECFC derived cells only. Vessel number (vessels/mm^2^) was estimated using Image J 1.38× software. Statistical analysis was performed using Student’s *t* test. (c) Vessel diameter estimation in matrigel implants containing SS-AF-MSCs, BMSCs or hDFs and UCB ECFC derived cells, SS-AF-MSCs only, or UCB ECFC derived cells only using Image J 1.38× software, (*p<0.05 Student’s *t* test). A minimum of 15 fields of view (40x) were analyzed from each photograph. Error bars indicate S.D. of the mean for 10 photographs from each group.

We next evaluated the potential of SS-AF-MSCs to support vessel formation in vivo. For this reason, we conducted experiments based on earlier studies [49], [54] with matrigel plugs containing: (i) UCB ECFC derived cells only, (ii) SS-AF-MSCs only, (iii) BMSCs only, (iv) hDFs only, (v) a mixture of SS-AF-MSCs and UCB ECFC derived cells, (vi) a mixture of BMSCs and UCB ECFC derived cells, or a (vii) mixture of hDFs and UCB ECFC derived cells. Matrigel plugs were harvested 2 weeks after implantation. In Figure 2a, a microscopic examination of a matrigel implant containing the SS-AF-MSCs and eGFP UCB ECFC derived cell mixture is presented, documenting the vessel network formed. Additionally, implants containing non-eGFP tagged human UCB ECFC derived cells and SS-AF-MSCs formed an extensive network of vessels (Figure 2 b to d) inosculating with the mouse vasculature. These vessels were successfully perfused as demonstrated after i.v. injection of tomato lectin (Figure 2 d). Vessel density in the implants was observed by staining sections for human and mouse CD31. Human CD31 was expressed ubiquitously in the matrigel area (Figure S4), whereas mouse CD31 was primarily found at the edge of the matrigel implant, where the human and mouse vascular network was connected. A similar vascular network was confirmed in the matrigel implants containing eGFP tagged UCB ECFC derived cells and SS-AF-MSCs (Figure 2 f). Vessels containing human CD31 expressing cells were not detected in any of the implants containing SS-AF-MSCs only (Figure 2 g), whereas, in implants containing UCB ECFC derived cells only (Figure 2 h), just a few superficial vessels were detected. The connection of human vessels to the murine vasculature is not only supported by the ability to perfuse the resultant human vessels with tomato lectin after its i.v. injection (Figure 2 d), but also by the demonstration of murine erythrocytes within human vessels positive for human CD31 antigen (Figure 3 a (i-iii) ). The numbers of vessels formed and the vessel diameters for single or combined cell populations transplanted in vivo in comparison with BMSCs and hDFs are shown in Figure 3 b and c. There was a statistical difference in vessel formation by UCB ECFC derived cells in the presence (59.1±12.7/mm^2^) compared to the absence of SS-AF-MSCs (11.1±0.4/mm^2^) (p<0.05 Student’s *t* test; n = 3, independent experiments), but no statistical difference in the diameters of the vessels which formed (11.7±1.9 and 13.4±2.6 microns respectively for the presence and absence of SS-AF-MSCs) (p>0.05 Student’s *t* test; n = 3 independent experiments). Notably, there was a statistically significant difference in vessel formation by UCB ECFC derived cells in the presence of SS-AF-MSCs (59.1±12.7/mm^2^) compared to BMSCs (42.2±4.9/mm^2^) (p<0.05 Student’s *t* test; n = 3, independent experiments). The average number of vessels formed in the presence of hDFs (47.8±6.4/mm^2^) differed from that in the presence of SS-AF-MSCs but this did not reach statistical significance (p = 0.12 Student’s *t* test; n = 3, independent experiments).

### SS-AF-MSCs Possess Distinctive Secretome Profiles Compared with BMSCs and hDFs

**Figure 4:**
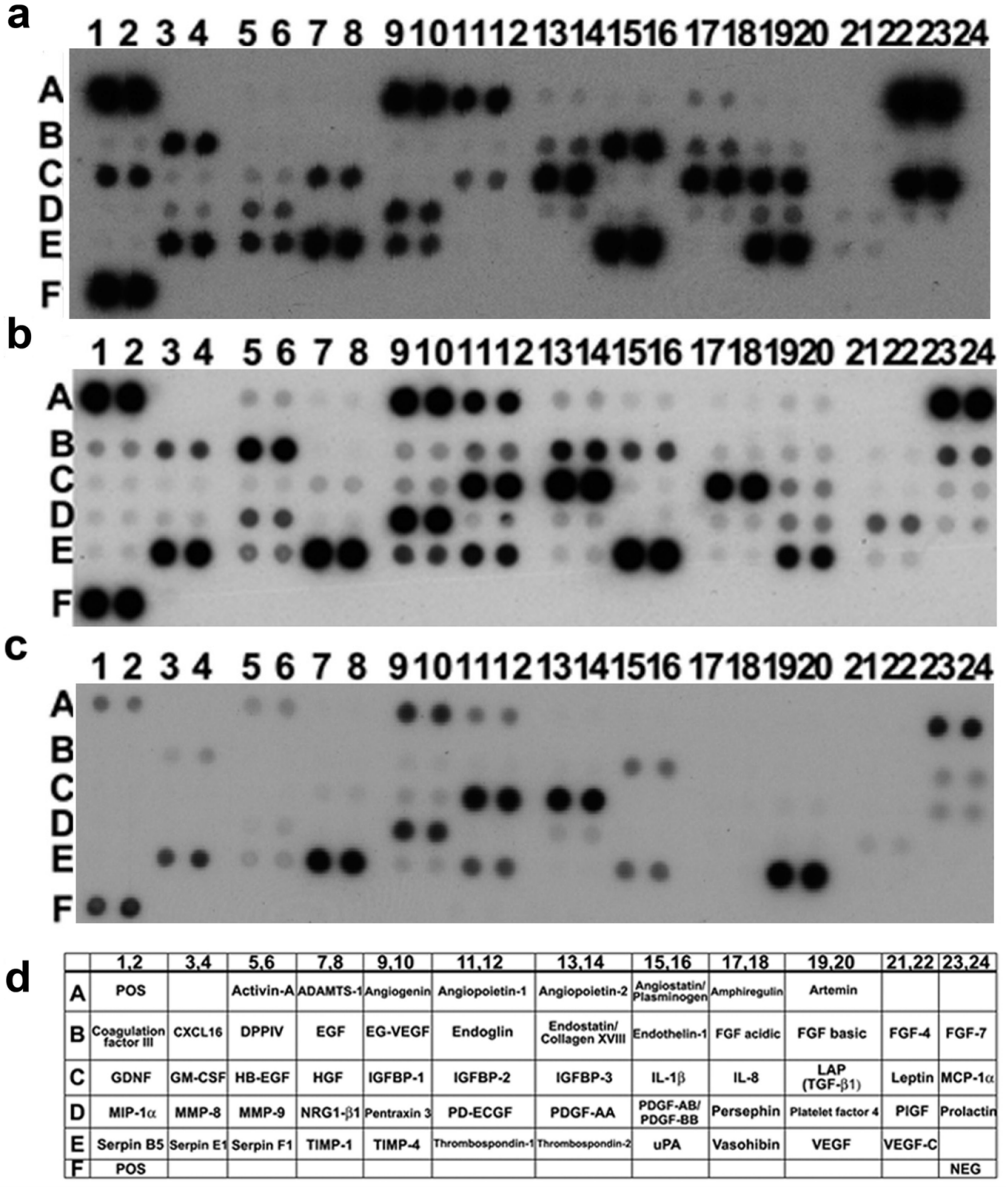
Analysis of angiogenic factors secreted by SS-AF-MSCs, BM-MSCs and hDFs in vitro using proteome arrays. (a-c) Representative proteome profiler arrays for (a) SS-AF-MSCs, (b) BMSCs and (c) hDFs respectively; (d) corresponding names of each molecule within the array summarized in tabular form.

**Figure 5:**
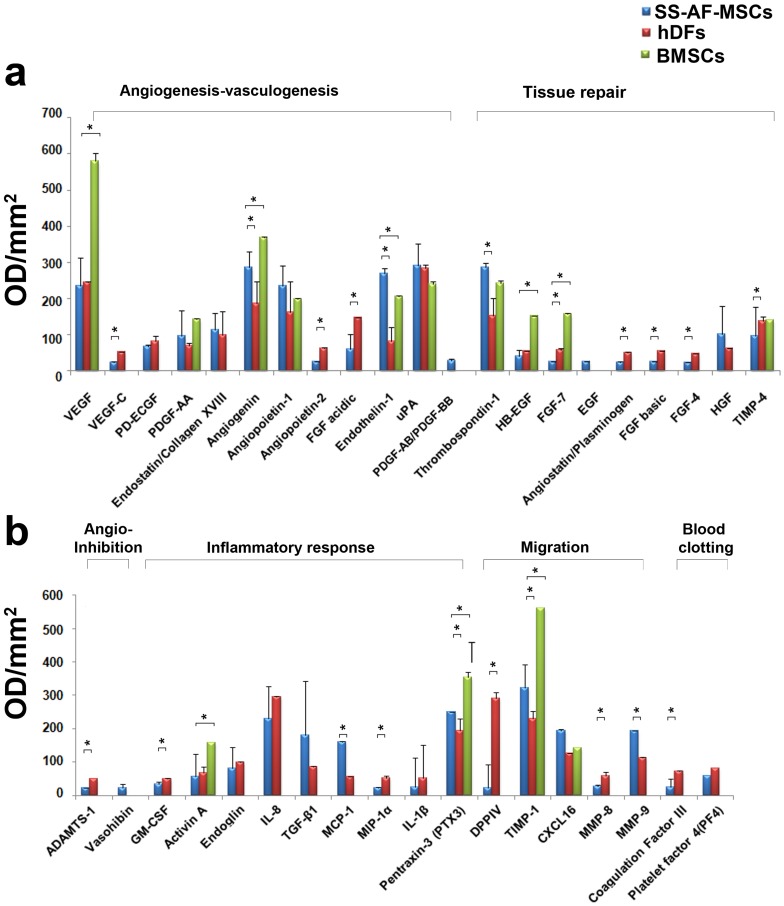
Secreted angiogenic factors in SS-AF-MSC-, BMSCs and hDF- conditioned media (CM) using proteome arrays. (a, b) Relative expression levels of angiogenic factors secreted from SS-AF-MSC-, BMSC- and hDF-CM according to their function. Values are normalized to positive controls. Values are means ± S.D. for three independent experiments, (*p<0.05 Student’s *t* test).

To define the profile of the molecular mediators of angiogenesis/vasculogenesis secreted by SS-AF-MSCs, we examined the conditioned medium (CM) derived from SS-AF-MSCs and directly compared this with BMSC-CM and hDF-CM using Proteome Profiler Human Arrays (see Materials and Methods and Figure 4, 5 and Table S1). Both 24 hour CM and sham media described above were incubated with arrays to allow detection of 55 secreted factors (Table S1). The most interesting factors detected were categorized into 6 groups according to information from the manufacturer and their known functions (Figure 5), although it should be noted that molecules may have multiple functions. Factors that regulate angiogenesis/vasculogenesis included VEGF, PDGF and angiopoietin family members, such as PDGF-AA, PDGF-AB/BB, angiopoietin-1, angiopoietin-2, VEGF, VEGF-C and PIGF as well as other such factors as endostatin, angiogenin and endothelin-1. Molecules such as uPA, thrombospondin-1, FGF-4, EGF, HB-EGF, angiostatin and HGF were categorized as the tissue repair group. ADAMTS-1 and vasohibin have been listed in the recent literature as angio-inhibitory molecules (angio-inhibition group), while activin A, endoglin, IL-8, TGF-β1, MCP-1, ΜIP-1α, IL-1β and pentraxin-3 were classified as the inflammatory response group. The migration group included molecules, such as DPPIV, TIMP-1, MMP-8 and MMP-9.

Interestingly, each cell population had a distinctivesecretome profile, although many of the same factors assayed were detected in both SS-AF-MSC-CM (55 factors) and hDF-CM (52 factors). Both SS-AF-MSCs and hDFs secreted some factors in similar relative amounts. These included angiopoietin-1, PD-ECGF, uPA and endostatin/collagen XVIII which facilitate angiogenesis (Figure 4 and 5 and Table S1). However, the commonly shared factors more often occurred in differing amounts in CM from each of the two cell types and more often at significantly higher levels in hDF-CM than in SS-AF-MSC-CM (Figure 5; Table S1). Indeed, almost 70% of the secreted factors, including VEGF-C, EG-VEGF, PIGF, angiostatin, angiopoietin-2, FGF-4, FGF-7, acidic and basic FGFs, GM-CSF, MIP-1α, IGBP-1, thrombospondin -2, leptin, ADAMTS-1, DPPIV, TIMP-4 and MMP-8, were found at relatively higher concentrations in hDF-CM than in SS-AF-MSC-CM (p<0.05 for all). In contrast, SS-AF-MSCs secreted endothelin-1, angiogenin, MMP-9, MCP-1, serpin-E1, TIMP-1 and thrombospondin-1 at relatively higher levels than hDFs (p<0.05 for all). The BMSC secretome was more limited than that found for SS-AF-MSCs and hDFs. Indeed, less than half the angiogenic factors detected in the SS-AF-MSC-CM and hDF-CM were found in human BMSC-CM (20 factors only) (Figure 4 and Table S1). Of note for the commonly secreted factors, activin-A, angiogenin, FGF7, HB-EGF, IGFBP-1, -2 and -3, pentraxin-3, PIGF, serpin-E1, TIMP-1 and VEGF were detected at significantly higher levels in BMSC-CM than in both SS-AF-MSC-CM and hDF-CM (p<0.05 for all; Figure 4, 5 and Table S1).

**Figure 6:**
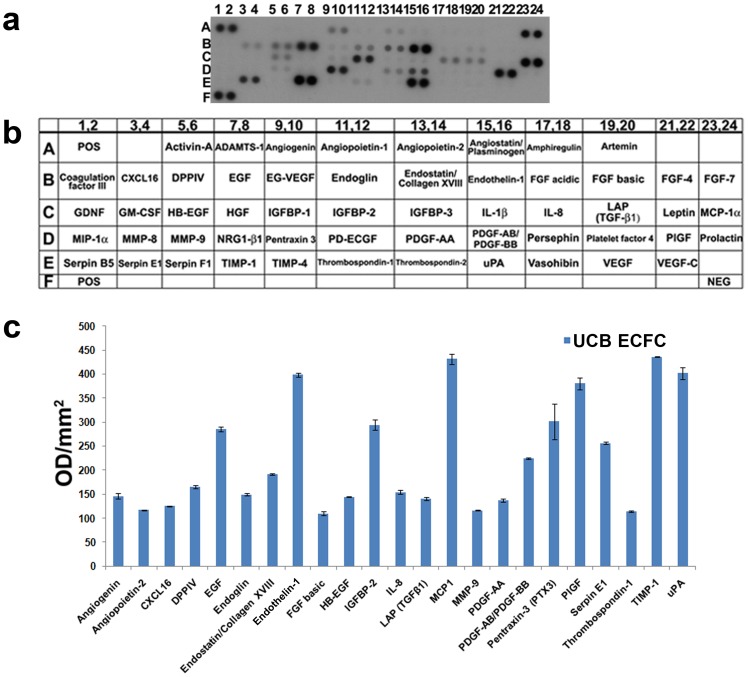
Analysis of angiogenic factors secreted by UCB ECFC derived cells in vitro. (a) Representative proteome profiler array for UCB ECFC derived cell-CM; (b) corresponding names of each molecule within the array summarized in tabular form; (c) Relative expression levels of angiogenic factors in UCB ECFC derived cell-CM. Values are normalized to positive controls. Values are means ± S.D. for three independent experiments, (*p<0.05 Student’s *t* test).

We next analyzed the factors present in UCB ECFC derived cell-CM identifying 23 secreted molecules, which included angiogenin, angiopoietin-2, CXCL16, DPPIV, EGF, endoglin, endostatin, endothelin-1, FGF basic, HB-EGF, IGFBP-2, IL-8, TGF-β1, MCP-1, MMP-9, PDGF-AA, PDGF-AB/BB, pentraxin-3, PIGF, serpin E1, thrompospodin-1, TIMP-1 and uPA (Figure 6). Of these, angiogenin, CXCL16, HB-EGF, endothelin-1, IGFBP-2, pentraxin-3, PIGF, serpin E1, TIMP-1, thrombospondin-1 and uPA were detected in both SS-AF-MSC-CM and BMSC-CM, while angiopoietin-2, DPPIV, EGF, endoglin, endostatin FGF-basic, IL-8, TGF-β1, MCP-1 and MMP-9 were found in both SS-AF-MSC-CM and hDF-CM.

Some factors secreted by SS-AF-MSCs were not detected in CM from other cell types. EGF and PDGF-AB/BB were found in SS-AF-MSC-CM, but not in the hDF-CM (Figure 4, 5 and Table S1). Notably, the BMSC-CM lacked many of the inflammatory response molecules found in SS-AF-MSC-CM and hDF-CM (Figure 5). Those factors not detected in the BMSC-CM when compared to SS-AF-MSC-CM and/or hDF-CM included both the inflammatory group (endoglin, IL-8, TGF-β1, MCP-1, MIP-1α, GM-CSF, IL-1β) and molecules in other categories (ADAMTS-1, angiopoietin-2, angiostatin, DDPIV, EGF, EG-VEGF, endostatin, HGF, IL-1β, MMP-8, MMP-9, PD-ECGF, PDGF-AB/BB, thrombospondin-2 and VEGF-C; Table S1). Of the 4 FGFs (FGF-acidic, -basic, -4 and -7) found in the SS-AF-CM and hDF-CM, only FGF-7 was detected in BMSC-CM. Those factors not detected in either BMSC-CM or UCB ECFC derived cell-CM, but which were present in SS-AF-MSC-CM or hDF-CM included ADAMTS-1, angiopoietin-2, angiostatin, EG-VEGF, FGF-acidic, FGF-4, GM-CSF, HGF, IL-1β, IL-8, MMP-8, PD-ECGF and VEGF-C (Figures 5, 6 and Table S1). Notably, when comparing hDFs, BMSCs, SS-AF-MSCs and UCB ECFC derived cells, vasohibin, which was categorised in the angio-inhibitor category, was the only factor exclusively secreted by SS-AF-MSCs in this analysis.

Therefore, we selected one factor from each of the three categories, angiogenesis/vasculogenesis, inflammatory response and migration, for further functional analyses since we did not detect these in BMSC-CM and since BMSC, on average, was the poorest supporter of vessel formation by UCB ECFC derived cells in vivo. These were PDGF-AB/BB, which was detected in SS-AF-MSC-CM and UCB ECFC-CM only, together with IL-8 and MMP9, which were present in all conditioned media except that from BMSCs (Figures 4 and 5).

### The Role of IL-8, PDGF-AB/BB and MMP9 Molecules in Modulating Migration, Proliferation and Vascular Tubule Formation of UCB ECFC Derived Cells

**Figure 7:**
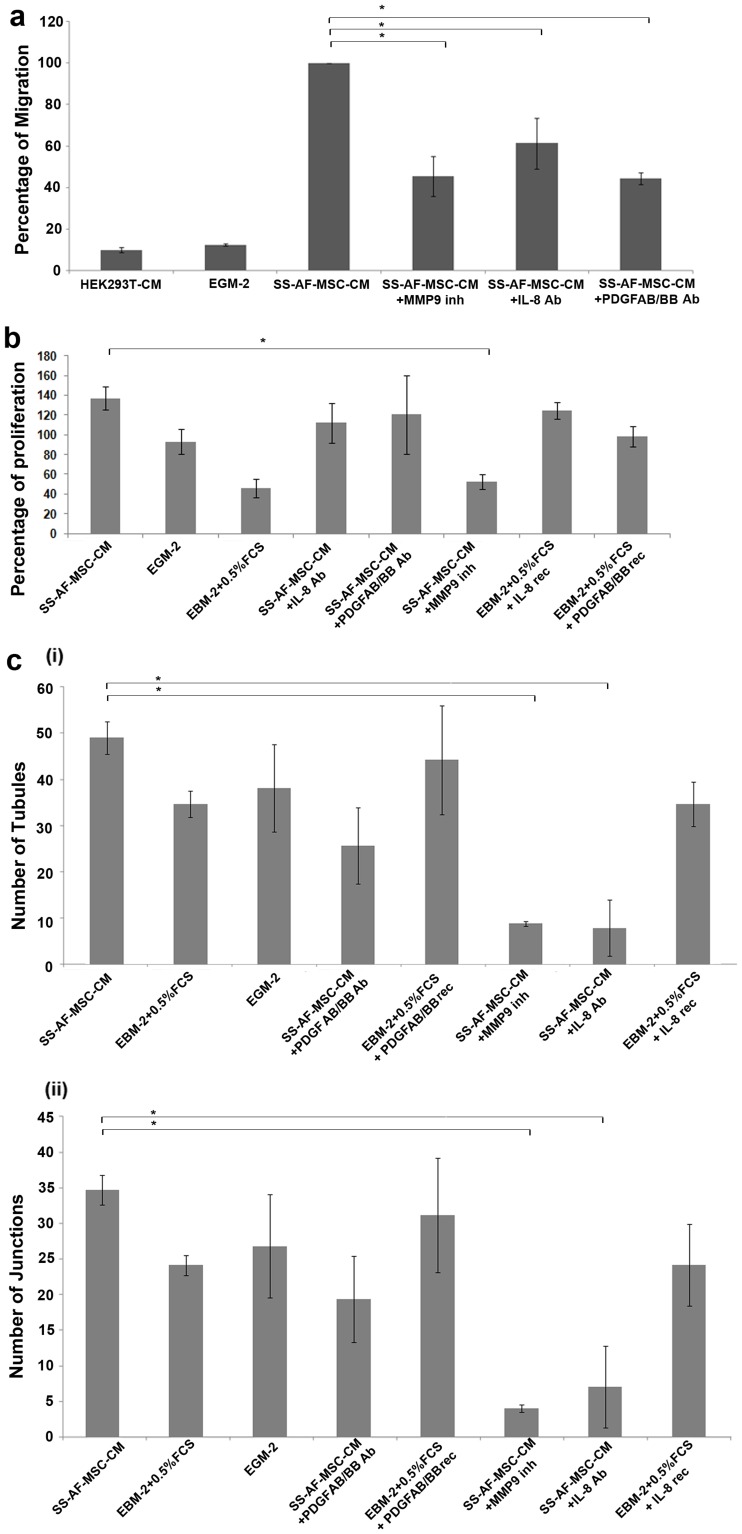
Role of IL-8, PDGF-AB/BB and MMP9 molecules from conditioned media in migration, proliferation and ability of tubule formation of UCB ECFC derived cells. (a) Histograms showing the migration of UCB ECFC derived cells towards SS-AF-MSC-conditioned medium (CM), EGM-2 medium, control medium (EBM-2, 0.5% (v/v) FCS), SS-AF-MSC-CM+IL8 neutralizing Ab, SS-AF-MSC-CM +PDGF-AB/BB neutralizing Ab or SS-AF-MSC-CM+MMP9 inhibitor (inh). Values are means ± S.D. for three independent experiments (*p<0.05 Student’s *t* test). (b) Examination of the proliferation rate in vitro of UCB ECFC derived cells under the same conditions. Control medium with recombinant (rec) IL-8 or PDGF-AB/BB was also included. Values are means ± S.D. for three independent experiments, (*p<0.05 Student’s *t* test). (c) In vitro angiogenesis matrigel assay for UCB ECFC derived cells under the respective conditions for estimation of the number of (i) tubules and (ii) junctions formed. Error bars indicate S.D. of the mean for 10 (5x) photographs from each group (*p<0.05 Student’s *t* test).

To confirm that highly secreted factors from SS-AF-MSCs, such as IL-8, PDGF-AB/BB and MMP9, function in regulating in UCB ECFC derived cell motility, an in vitro transwell migration assay was performed using neutralizing Abs for the IL-8 and PDGF-AB/BB cytokines and an inhibitor for MMP-9. The addition of IL-8 and PDGFAB/BB neutralizing Abs to the SS-AF-MSC-CM (Figure 7a) resulted in a respective 61.47±12.53% and 44.36±2.81% reduction in the migration of UCB ECFC derived cells. A similar effect was observed when the MMP-9 inhibitor was added to the SS-AF-MSC CM (Figure 7a), with a reduction of 63.95±7.09% in the UCB ECFC derived cell migration. We next examined the effect of SS-AF-MSC-CM on the proliferation of UCB ECFC derived cells and showed that the presence of MMP-9 inhibitor in SS-AF-MSC-CM resulted in a statistically significant decrease in the proliferation of UCB ECFC derived cells (52.7±7.7%, *p*<0.05, Student’s *t*-test, Figure 7b).

SS-AF-MSC-CM also facilitated vascular tubule formation by UCB ECFC derived cells in the matrigel assay in vitro (Figure 7c). Furthermore, following addition of IL-8 neutralizing Ab or MMP-9 inhibitor to the SS-AF-MSC-CM, UCB-ECFC derived cells exhibited impaired tubule formation with reduced numbers of junctions (Figure 7c i and ii). PDGF-AB/BB inhibition showed a reduced but not a statistically significant effect on UCB ECFC derived cell tubule formation (Figure 7c i and ii).

## Discussion

Recent studies have reported that human BMSCs and adipose tissue (AT) derived MSCs can support human blood vessel formation in vivo in animal models when they are co-transplanted with human blood ECFC derived cells [54]–[56]. In the studies presented here, we show that SS-AF-MSCs have the added value of also supporting new vessel formation both in vitro and in vivo, but notably they provided better support for angiogenesis/vasculogenesis in an in vivo NOD-SCID murine model than BMSCs did, which had been pre-selected for their ability to optimally support vascular tubule formation in vitro.

There are several potential explanations for these in vivo observations. The first is that MSCs at earlier stages of ontogeny have increased potency compared to adult MSCs in dampening a host versus graft response and/or in supporting stable vessel formation in vivo. It has, for example, been shown that fetal MSCs, such as those derived from second trimester amniotic fluid, exert stronger immunomodulatory effects in terms of transplantation tolerance than adult MSCs [57]. The enhanced immunomodulatory properties of SS-AF-MSCs may thus permit better vessel formation by human UCB ECFC derived cells when compared with BMSCs in our NOD-SCID mouse xenograft model, which lacks T and B lymphoid cells, but not NK cells [58]. These mice are known to be a less efficient model for reconstituting human hematopoiesis than NSG (NOD/Lt-scid/IL2Rgamma (null)) mice, which lack NK cells and demonstrate other defects in innate immunity not observed in the NOD-SCID mouse strain [58], [59]. However, as the NSG xenograft model has not to our knowledge been tested in vasculogenesis assays in vivo, future studies would need to determine if NSG mice provide a more efficient in vivo readout of vasculogenesis than those currently in use (athymic nu/nu, SCID or NOD-SCID mice) and which are not NK cell deficient [59]. While the use of the NOD-SCID in vivo model and enhanced immunomodulatory capability of fetally derived MSCs might partially explain why SS-AF-MSCs are more efficient in promoting vasculogenesis in vivo than BMSCs, this does not explain why the hDFs more closely resemble SS-AF-MSCs in supporting more vessel formation in vivo than BMSCs do. While SS-AF-MSCs and BMSCs both possess immunomodulatory activities (reviewed in [5], [9], [26]), this is not the case for hDFs and these latter cells would therefore be expected to be less well tolerated in in vivo NOD-SCID xenografts.

An alternative explanation and perhaps a more significant contributory effect for achieving more efficient in vivo vasculogenesis could relate to the balance of angiogenic factors secreted by the supporting hDFs or MSCs. Indeed, our studies demonstrate that, although SS-AF-MSCs, BMSCs and hDFs all promoted vessel formation, significantly more vessels formed in vivo in the presence of SS-AF-MSCs and hDFs than in the presence of BMSCs, and each displayed unique secretome profiles. Notably, BMSCs secreted less than half the angiogenic factors detected in conditioned media derived from SS-AF-MSCs and hDFs, while SS-AF-MSCs and hDFs secreted many factors in common. Those factors present in both SS-AF-MSC-CM and hDF-CM, but not detectable in BMSC-CM, included IL-8, FGF-4, angiopoietin-2, HGF, PD-ECGF and MMP-9, while SS-AF-MSCs also secreted PDGF-AB/BB. Each of these factors has defined roles in regulating angiogenesis and vasculogenesis [53], [60]–[69], and our studies confirmed that IL-8, MMP-9 and PDGF-AB/BB secreted by the SS-AF-MSCs were functional in promoting UCB ECFC derived cell migration, while IL-8 also promoted UCB ECFC derived cell proliferation. Interestingly, recent studies from Kachgal and Putnam [61] demonstrate that adipose tissue (AT) MSCs resemble human lung fibroblasts more closely than BMSCs in their angiogenic factor profile, with uPA and MMPs in particular playing key but distinct roles in promoting vessel formation and integrity. Other studies identify leptin as an angiogenic factor in AT-MSCs [65]. As with our own studies, this suggests that different MSCs/perivascular cells promote vessel formation by differing mechanisms and that MSCs derived from bone marrow may differ significantly in their angiogenic profiles from those found in amniotic fluid or adipose tissue. Although beyond the scope of this study, understanding these mechanisms in more detail should prove beneficial not only for our understanding of vessel formation during development and within specific microenvironmental niches, but also for sourcing cells or defining combinations of factors for vascular repair, for revascularizing damaged tissues or organs and for promoting neovascularization in engineered tissues.

In our previous studies, we successfully isolated and expanded over many passages (at least 40) MSCs from human second trimester amniotic fluid [29], [30]. Indeed, we and others have demonstrated that SS-AF-MSCs can be expanded and produced rapidly to a clinical scale while retaining their immunosuppressive properties [30], [70]. Such AF-MSCs exhibit chromosomal stability with no karyotypic abnormalities detected even at high passages, express pluripotent markers, possess long telomeres and do not form tumours in vivo [11], [21], [23], [29], [30]. As putative intermediates in terms of their potentiality between human embryonic or induced pluripotent stem (ES/iPS) cells and postnatal MSCs and without the inherent difficulties that ES/iPS cells have in forming spontaneous tumors in vivo or the lower proliferative and hence expandability difficulties of adult BMSCs [8], [26], the AF-MSCs constitute a very interesting and promising population of cells for clinical revascularization. One disadvantage of these AF-MSCs is their collection at the time of amniocentesis, which is not routinely performed in all pregnancies and which can rarely lead to fetal loss. However, where amniotic fluid is collected for routine prenatal diagnosis and by taking advantage of protocols developed from our work and our preclinical studies [29], [30], [37], [45], excess amniotic fluid that is normally discarded can be used to source SS-AF-MSCs, which can then generate millions of cells in culture in a relatively short period of time, enough for prospective clinical applications. Thus, amniotic fluid may prove a valuable source of MSCs for future autologous or allogeneic clinical applications related to tissue revascularization and repair.

## Supporting Information

Figure S1 **Phenotype of umbilical cord blood endothelial colony forming cell (ECFC) derived cells.** Representative FACS histograms of UCB ECFC derived cells at passage 3–4. Each specifically fluorescently tagged isotype control or biomarker monoclonal antibody used is listed below the relevant histogram. Values are means of median fluorescence intensities (MFI)±S.E.M. for n = 3 independent batches of cells
(TIF)

Figure S2 **SU6668 treatment reduces neovascularization in vitro.** (a-c) Representative fields of vascular tubules after 14 days in culture and chronically exposed to control conditions (no drug), 5 µM and 10 µM SU6668 inhibitor. Scale bar = 500 µm. (d-f) Quantification of vascular tubule phenotypes at day 14 with and without exposure to SU6668 inhibitor. There is a significant reduction in the number of junctions, tubules and total tubule length following exposure to 5 µM and 10 µM SU6668 inhibitor compared to control conditions (*p<0.05 Student’s *t* test). Error bars are means±S.D. for three independent experiments.
(TIF)

Figure S3 **Proliferation assay for SS-AF-MSCs, BMSCs, hDFs and UCB ECFC derived cells in presence of SU6668.** Percentage of proliferation increase of SS-AF-MSCs, BMSCs, DFs and UCB ECFC derived cells in presence of 5 µΜ and 10 µΜ SU6668 3 (i) and 8 (i) days after treatment, Cells cultured in absence of SU6668 were used as control (untreated). Values are means (MFI)±S.E.M. for n = 3 independent batches of cells. There was no significant difference for each group in the presence or absence of SU6668 (p>0.05 Student’s *t* test.
(TIF)

Figure S4 **Immunofluorescence vessel imaging in matrigel implants in vivo at 40× magnification.** Representative photomicrographs (i-iii) of the matrigel implants containing UCB ECFC derived cells and SS-AF-MSCs stained for hCD31 (green) and DAPI (blue) at 40× magnification. hCD31 staining is localized at the cell membrane (i and iii).
(TIF)

Table S1 **Summary of Angiogenic Growth Factors and Cytokines Secreted by SS-AF-MSCs, BMSCs and hDFs.**
(DOC)
