# Supplementary figures and images for: Spindle Shaped Human Mesenchymal Stem/Stromal Cells from Amniotic Fluid Promote Neovascularization
Source: PLoS One. 2013 Jan 24;8(1):e54747. doi: 10.1371/journal.pone.0054747 (PMC3554641; doi:10.1371/journal.pone.0054747)

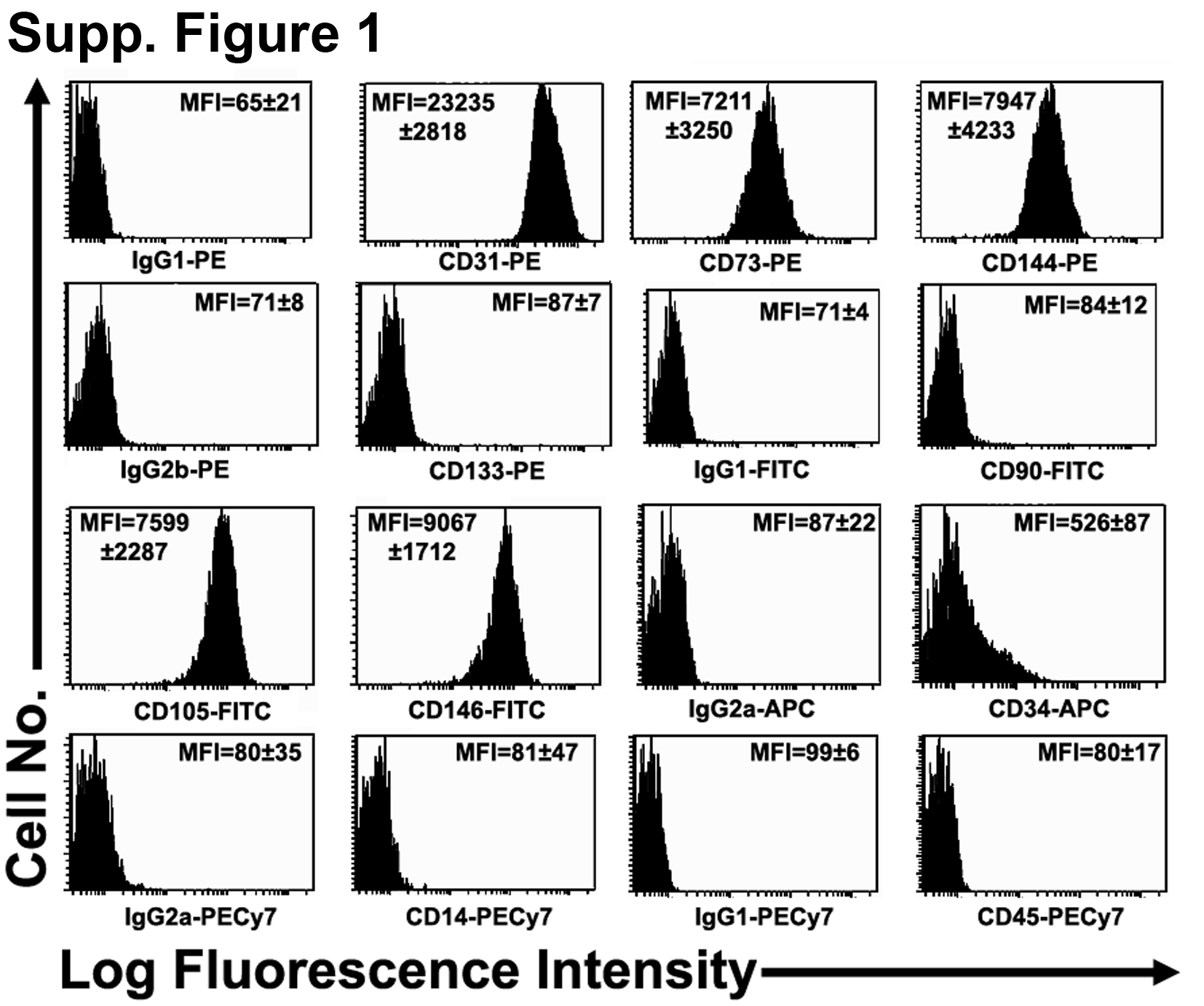

Supplement: Figure S1 — Phenotype of umbilical cord blood endothelial colony forming cell (ECFC) derived cells. Representative FACS histograms of UCB ECFC derived cells at passage 3–4. Each specifically fluorescently tagged isotype control or biomarker monoclonal antibody used is listed below the relevant histogram. Values are means of median fluorescence intensities (MFI)±S.E.M. for n = 3 independent batches of cells (TIF) [file pone.0054747.s001.tif]

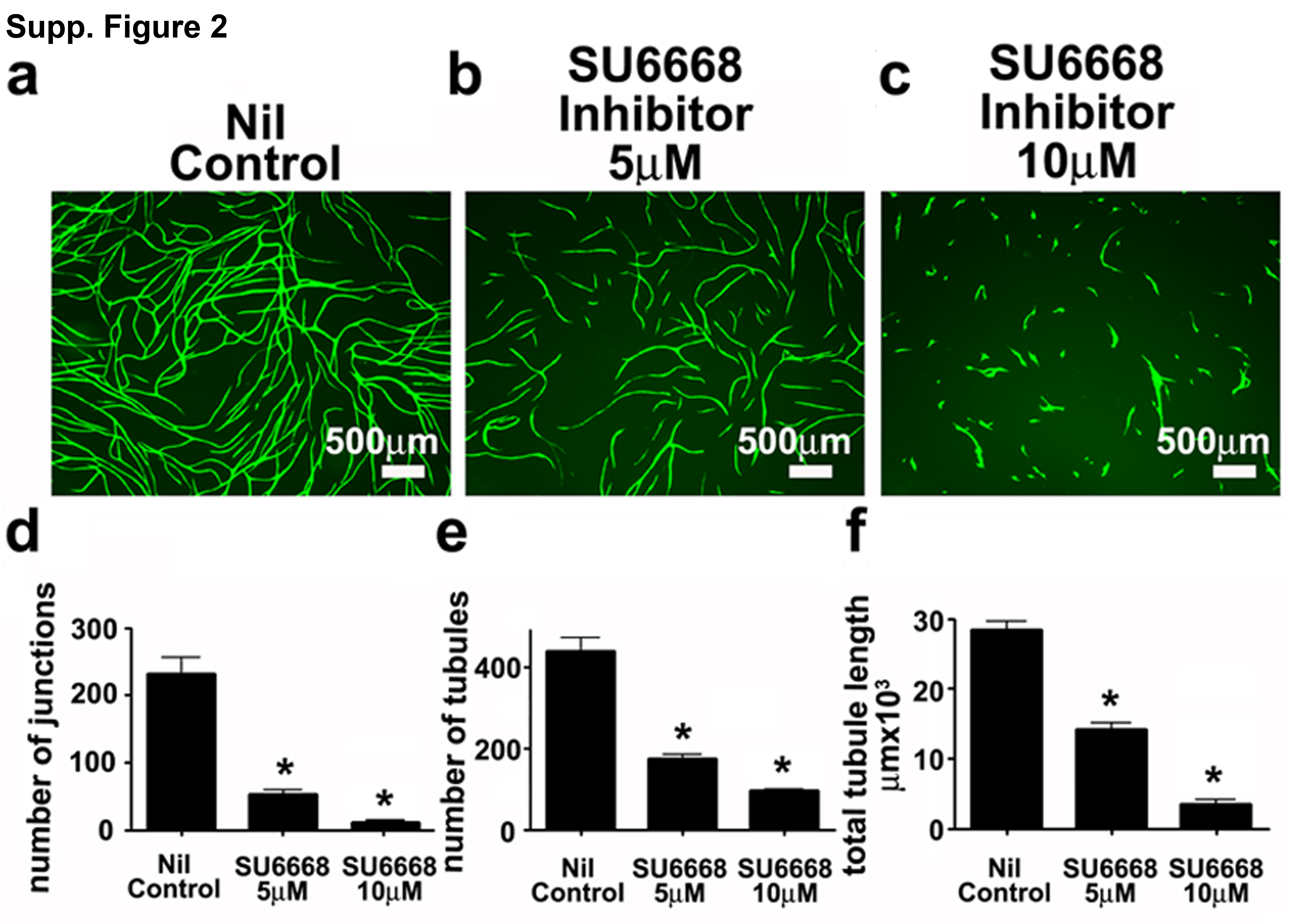

Supplement: Figure S2 — SU6668 treatment reduces neovascularization in vitro. (a-c) Representative fields of vascular tubules after 14 days in culture and chronically exposed to control conditions (no drug), 5 µM and 10 µM SU6668 inhibitor. Scale bar = 500 µm. (d-f) Quantification of vascular tubule phenotypes at day 14 with and without exposure to SU6668 inhibitor. There is a significant reduction in the number of junctions, tubules and total tubule length following exposure to 5 µM and 10 µM SU6668 inhibitor compared to control conditions (*p<0.05 Student’s t test). Error bars are means±S.D. for three independent experiments. (TIF) [file pone.0054747.s002.tif]

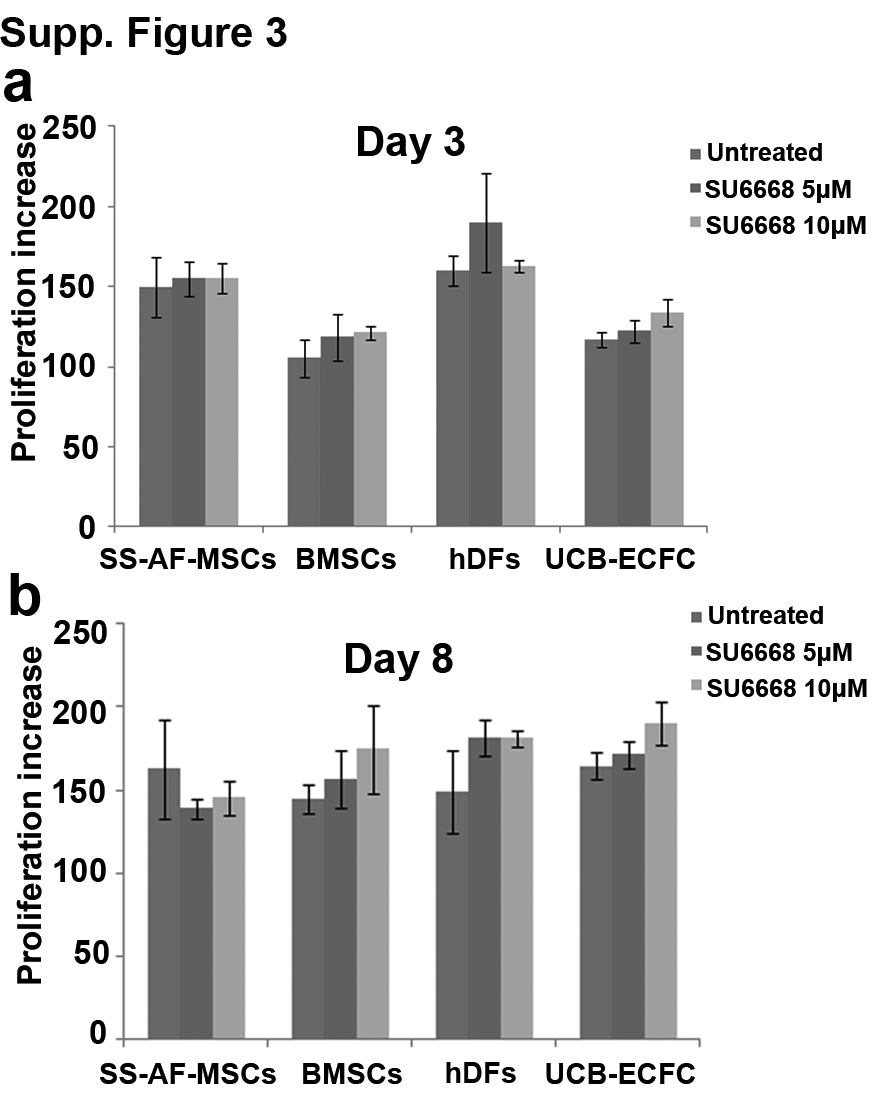

Supplement: Figure S3 — Proliferation assay for SS-AF-MSCs, BMSCs, hDFs and UCB ECFC derived cells in presence of SU6668. Percentage of proliferation increase of SS-AF-MSCs, BMSCs, DFs and UCB ECFC derived cells in presence of 5 µΜ and 10 µΜ SU6668 3 (i) and 8 (i) days after treatment, Cells cultured in absence of SU6668 were used as control (untreated). Values are means (MFI)±S.E.M. for n = 3 independent batches of cells. There was no significant difference for each group in the presence or absence of SU6668 (p>0.05 Student’s t test. (TIF) [file pone.0054747.s003.tif]

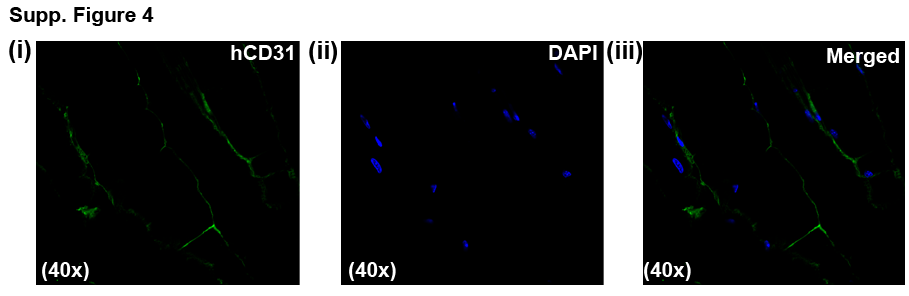

Supplement: Figure S4 — Immunofluorescence vessel imaging in matrigel implants in vivo at 40× magnification. Representative photomicrographs (i-iii) of the matrigel implants containing UCB ECFC derived cells and SS-AF-MSCs stained for hCD31 (green) and DAPI (blue) at 40× magnification. hCD31 staining is localized at the cell membrane (i and iii). (TIF) [file pone.0054747.s004.tif]
