# Supplementary material for: Spindle Shaped Human Mesenchymal Stem/Stromal Cells from Amniotic Fluid Promote Neovascularization
Source: PLoS One. 2013 Jan 24;8(1):e54747. doi: 10.1371/journal.pone.0054747 (PMC3554641; doi:10.1371/journal.pone.0054747)
Supplement: Table S1 — Summary of Angiogenic Growth Factors and Cytokines Secreted by SS-AF-MSCs, BMSCs and hDFs. (DOC) [file pone.0054747.s005.doc]

**Supplementary Table 1: Summary of** Angiogenic Growth Factors and Cytokines Secreted by SS-AF-MSCs, BMSCs and hDFs.

| **CYTOKINES** | **SS-AF-MSCs** | **hDFs** | **t-Test** | **SS-AF-MSCs** | **BMSCs** | **t-Test** | **hDFs** | **BMSCs** | **t-Test** |
| --- | --- | --- | --- | --- | --- | --- | --- | --- | --- |
| **Activin A** | + | + |  | + | ↑ | * | + | ↑ | * |
| **ADAMTS-1** | + | ↑ | * | + | - |  | + | - |  |
| **Angiogenin** | ↑ | + | * | + | ↑ | * | + | ↑ | * |
| **Angiopoietin-1** | + | + |  | + | + |  | + | + |  |
| **Angiopoietin-2** | + | ↑ | * | + | - |  | + | - |  |
| **Angiostatin/Plasminogen** | + | ↑ | * | + | - |  | + | - |  |
| **Amphiregulin** | + | ↑ | * | + | - |  | + | - |  |
| **Artemin** | + | + |  | + | - |  | + | - |  |
| **Coagulation Factor III** | + | ↑ | * | + | - |  | + | - |  |
| **CXCL16** | + | + |  | + | + |  | + | + |  |
| **DPPIV** | + | ↑ | * | + | - |  | + | - |  |
| **EGF** | + | - |  | + | - |  | - | - |  |
| **EG-VEGF** | + | ↑ | * | + | - |  | + | - |  |
| **Endostatin/**  **Collagen XVIII** | + | + |  | + | - |  | + | - |  |
| **Endothelin-1** | ↑ | + | * | ↑ | + | * | + | ↑ | * |
| **Endoglin** | + | + |  | + | - |  | + | - |  |
| **FGF-7** | + | ↑ | * | + | ↑ | * | + | ↑ | * |
| **FGF acidic** | + | ↑ | * | + | - |  | + | - |  |
| **FGF basic** | + | ↑ | * | + | - |  | + | - |  |
| **FGF-4** | + | ↑ | * | + | - |  | + | - |  |
| **GDNF** | + | + |  | + | - |  | + | - |  |
| **GM-CSF** | + | ↑ | * | + | - |  | + | - |  |
| **HB-EGF** | + | + |  | + | ↑ | * | + | ↑ | * |
| **HGF** | + | + |  | + | - |  | + | - |  |
| **IGFBP-1** | + | ↑ | * | + | ↑ | * | + | ↑ | * |
| **IGFBP-2** | + | + |  | + | ↑ | * | + | ↑ | * |
| **IGFBP-3** | + | + |  | + | ↑ | * | + | ↑ | * |
| **IL-1β** | + | + |  | + | - |  | + | - |  |
| **IL-8** | + | + |  | + | - |  | + | - |  |
| **TGF-β1** | + | + |  | + | - |  | + | - |  |
| **Leptin** | + | ↑ | * | + | - |  | + | - |  |
| **MCP-1** | ↑ | + | * | + | - |  | + | - |  |
| **MIP-1α** | + | ↑ | * | + | - |  | + | - |  |
| **MMP-8** | + | ↑ | * | + | - |  | + | - |  |
| **MMP-9** | ↑ | + | * | + | - |  | + | - |  |
| **NRG1-β1** | + | + |  | + | - |  | + | - |  |
| **Pentraxin-3 (PTX3)** | + | + |  | + | ↑ | * | + | ↑ | * |
| **PD-ECGF** | + | + |  | + | - |  | + | - |  |
| **PDGF-AA** | + | + |  | + | + |  | + | ↑ | * |
| **PDGF-AB/PDGF-BB** | + | - |  | + | - |  | - | - |  |
| **Persefin** | + | ↑ | * | + | - |  | + | - |  |
| **Platelet factor 4(PF4)** | + | ↑ | * | + | - |  | + | - |  |
| **PlGF** | + | ↑ | * | + | ↑ | * | + | ↑ | * |
| **Prolactin** | + | ↑ | * | + | - |  | + | - |  |
| **Serpin B5** | + | ↑ | * | + | - |  | + | - |  |
| **Serpin E1** | ↑ | + | * | + | ↑ | * | + | ↑ | * |
| **Serpin F1** | + | + |  | + | + |  | + | + |  |
| **TIMP-1** | ↑ | + | * | + | ↑ | * | + | ↑ | * |
| **TIMP-4** | + | ↑ | * | + | + |  | + | + |  |
| **Thrombospondin-1** | ↑ | + | * | + | + |  | + | ↑ | * |
| **Thrombospondin-2** | + | ↑ | * | + | - |  | + | - |  |
| **uPA** | + | + |  | + | + |  | ↑ | + | * |
| **Vasohibin** | + | - |  | + | - |  | - | - |  |
| **VEGF** | + | + |  | + | ↑ | * | + | ↑ | * |
| **VEGF-C** | + | ↑ | * | + | - |  | + | - |  |

Summary of angiogenic factors detected in SS-AF-MSC-, hDF- and BMSC-CM. Experiments were performed in triplicate, (+: presence, ↑: statistically significant increased expression, - no detectable expression), (* p<0.05 Student’s *t* test).
